# Long-Term Effects of Postearthquake Distress on Brain Microstructural Changes

**DOI:** 10.1155/2014/180468

**Published:** 2014-01-14

**Authors:** Atsushi Sekiguchi, Yuka Kotozaki, Motoaki Sugiura, Rui Nouchi, Hikaru Takeuchi, Sugiko Hanawa, Seishu Nakagawa, Carlos Makoto Miyauchi, Tsuyoshi Araki, Atsushi Sakuma, Yasuyuki Taki, Ryuta Kawashima

**Affiliations:** ^1^Division of Medical Neuroimage Analysis, Department of Community Medical Supports, Tohoku Medical Megabank Organization, Tohoku University, 4-1 Seiryo-machi, Aoba-ku, Sendai 980-8575, Japan; ^2^Department of Functional Brain Imaging, Institute of Development, Aging and Cancer (IDAC), Tohoku University, 4-1 Seiryo-machi, Aoba-ku, Sendai 980-8575, Japan; ^3^Department of Advanced Brain Science, Smart Ageing International Research Center, IDAC, Tohoku University, 4-1 Seiryo-machi, Aoba-ku, Sendai 980-8575, Japan; ^4^International Research Institute of Disaster Science, Tohoku University, 4-1 Seiryo-machi, Aoba-ku, Sendai 980-8575, Japan; ^5^Division of Developmental Cognitive Neuroscience, IDAC, Tohoku University, 4-1 Seiryo-machi, Aoba-ku, Sendai 980-8575, Japan; ^6^Graduate Schools for Law and Politics, The University of Tokyo, 7-3-1 Hongo, Bunkyo-ku, Tokyo 113-0033, Japan; ^7^Department of Psychiatry, Tohoku University Graduate School of Medicine, 1-1 Seiryo-machi, Aoba-ku, Sendai 980-8574, Japan; ^8^Department of Nuclear Medicine and Radiology, IDAC, Tohoku University, 4-1 Seiryo-machi, Aoba-ku, Sendai 980-8575, Japan

## Abstract

Stressful events can have both short- and long-term effects on the brain. Our recent investigation identified short-term white matter integrity (WMI) changes in 30 subjects soon after the Japanese earthquake. Our findings suggested that lower WMI in the right anterior cingulum (Cg) was a pre-existing vulnerability factor and increased WMI in the left anterior Cg and uncinate fasciculus (Uf) after the earthquake was an acquired sign of postearthquake distress. However, the long-term effects on WMI remained unclear. Here, we examined the 1-year WMI changes in 25 subjects to clarify long-term effects on the WMI. We found differential FAs in the right anterior Cg, bilateral Uf, left superior longitudinal fasciculus (SLF), and left thalamus, suggesting that synaptic enhancement and shrinkage were long-term effects. Additionally, the correlation between psychological measures related to postearthquake distress and the degree of WMI alternation in the right anterior Cg and the left Uf led us to speculate that temporal WMI changes in some subjects with emotional distress occurred soon after the disaster. We hypothesized that dynamic WMI changes predict a better prognosis, whereas persistently lower WMI is a marker of cognitive dysfunction, implying the development of anxiety disorders.

## 1. Introduction

Stressful events have both short- and long-term effects on the brain [1, 2]. Acute and chronic stress-induced brain microstructural changes have been observed in prefrontal areas in rodents [3]. Recent human studies identified white matter microstructural changes due to stress using diffusion tensor imaging (DTI) methods [4] in subjects with posttraumatic stress disorder (PTSD) [5–8] as well as healthy survivors of a disaster [9]. These studies revealed lower white matter integrity (WMI) in several brain regions, including the cingulum (Cg) and uncinate fasciculus (Uf), in subjects who developed PTSD [5–8] (i.e., long-term effect) and in individuals soon after a disaster [9] (i.e., short term effect). However, because these previous studies employed cross-sectional designs, longitudinal WMI changes within individuals remained unclear.

Our previous longitudinal investigation unveiled the causal relationships between WMI changes and psychological distress soon after a disaster [10]. In our previous study, we collected DTI data from a group of healthy subjects before the Japanese earthquake (pre). Then, we recruited 30 subjects (male/female = 24/6, age = 21.0 ± 1.6 yr, range = 19 to 25 yr) from this group and examined results from DTI and from psychological measures related to postearthquake distress 3 to 4 months after the earthquake (post) to examine short-term effects. We found that lower WMI in the right anterior Cg before the earthquake was a preexisting vulnerability factor for postearthquake distress, and that increased WMI in the left anterior Cg and Uf after the earthquake was an acquired sign of post-earthquake distress [10].

In the current study, we examined WMI changes in subjects from the previous investigation 1 year later (followup) [10]. We tried to identify WMI changes that occurred in early (pre to post) and late (post to followup) phases after this stressful life event and investigated when and where these WMI changes occurred. In particular, we focused on the prognosis of FA changes in the right anterior Cg and the left anterior Cg and Uf, which were identified as a preexisting vulnerability factor and an acquired sign of post-earthquake distress, respectively.

## 2. Materials and Methods

### 2.1. Subjects

All subjects participated in our previous investigation [10, 11]. Of the 30 subjects in our previous DTI study [10], we rerecruited 25 subjects (male/female = 19/6, age = 21.7 ± 1.4 yr) and assessed their structural DTI results one year after the earthquake. We screened for neuropsychiatric disorders using the Mini International Neuropsychiatric Interview (M.I.N.I.) [12, 13]. Handedness was assessed using the Edinburgh Handedness Inventory [14]. All subjects provided written informed consent before participating in the current study, which examined the possible effects of psychological trauma on brain structure, in accordance with the Declaration of Helsinki [15]. The M.I.N.I. confirmed that no subject had any history of psychiatric illness including PTSD and no subjects were exposed to life-threatening experiences due to the earthquake or tsunami. The current study was approved by the Ethics Committee of Tohoku University.

### 2.2. Psychological Evaluations

All participants were interviewed by trained psychologists using the Japanese version of the clinician-administered PTSD scale (CAPS) structured interview [16, 17]. In accordance with the M.I.N.I., no subject was diagnosed as having PTSD. Levels of anxiety and depression were evaluated using the State-Trait Anxiety Inventory (STAI) [18, 19] and the Center for Epidemiologic Studies Depression Scale (CES-D) [20, 21]. Psychological traits related to resilience in response to stressful life events were assessed using the Japanese version of the Posttraumatic Growth Inventory (PTGI-J) [22, 23] and the Japanese version of the Rosenberg Self-Esteem Scale [24, 25]. All psychological measures were assessed at 3 to 4 months (post) and at 1 year (followup) after the earthquake.

### 2.3. Image Acquisition

All MRI data were acquired with a 3-T Philips Intera Achieva scanner. The diffusion-weighted data were acquired using a spin-echo EPI sequence (TE = 55 ms, FOV = 22.4 cm, 2 × 2 × 2 mm^3^ voxels, 60 slices). The diffusion weighting was isotropically distributed along 32 directions (*b* value = 1,000 s/mm^2^). Additionally, a dataset with no diffusion weighting (*b* value = 0 s/mm^2^; b0 image) was acquired. The total scan time was 7 min 17 s. Then, fractional anisotropy (FA) values were calculated from the collected images. This information is of particular interest when making inferences regarding white matter microstructural properties, as diffusion is faster along axons than in the perpendicular direction. Consequently, diffusion in white matter is anisotropic (i.e., diffusion rates in different directions are unequal). By contrast, isotropic diffusion is equally fast in all directions. FA in each voxel was used as a measure of the degree of diffusion anisotropy. FA varies between 0 and 1, with 0 representing isotropic diffusion and 1 representing diffusion occurring entirely in one direction. After DTI image acquisition, FA map images were calculated from DTI using software preinstalled on the Philips MR console.

### 2.4. Preprocessing of Diffusion Imaging Data

Preprocessing and data analysis were performed using statistical Parametric Mapping software (SPM5; Wellcome Department of Cognitive Neurology, London, UK) implemented in MATLAB (MathWorks, Natick, MA, USA). First, our original b0 image template was created as follows. Using the affine and nonlinear spatial normalization algorithm, the b0 images from the pre-earthquake scans of all subjects in this study were spatially normalized to the SPM5 T2 template, which is based on averages taken from 152 brains from the Montreal Neurological Institute database. Then, we calculated a mean image of the normalized b0 images as our original b0 image template. Using the affine and nonlinear spatial normalization algorithm, the b0 image of each participant was normalized to our original b0 image template. Before normalization of the FA map, the postearthquake FA maps were coregistered with the pre-earthquake FA maps from each subject. Then, using the parameter for this affine and nonlinear normalization procedure, an FA map image of each participant was spatially normalized to yield images with 2 × 2 × 2 mm voxels and spatially smoothed using a Gaussian kernel of 10 mm FWHM. The resulting maps representing FA were then subjected to the group regression analysis described below.

### 2.5. Statistical Analysis

Differences in FA between before the earthquake (pre), 3-4 months after the earthquake (post), and 1 year after the earthquake (followup) were compared using analysis of covariance (ANCOVA) in SPM5. The analysis was performed with sex and period between MR acquisition and the earthquake as additional covariates. Differential FA between time periods was detected as a main effect (pre/post/followup) using *F*-contrasts in SPM. The significance level was set at *P* = 0.05, corrected for multiple comparisons (voxel-level family-wise error) and *k* > 10 to suppress the possibility of small clusters arising by chance. Additionally, to check for structural changes between each period (pre versus post, pre versus followup, and post versus followup), paired *t*-tests were performed for each cluster identified as a main effect in the ANCOVA. Finally, to ascertain the 1-year prognosis of FA changes as a preexisting vulnerability factor and as an acquired sign of postearthquake distress, *post hoc* correlation analysis was performed including the scores for postearthquake distress (e.g., CAPS and STAI-state at post) and FA changes from pre to followup in the right anterior Cg (i.e., a preexisting vulnerability factor at Pre) as well as from post to followup in the left anterior Cg and Uf (i.e., an acquired sign at Post) within the clusters detected by the ANCOVA.

All FA tests were performed using an absolute threshold of FA >0.2 [26], such that if a voxel anywhere in the brain had an FA value >0.2 in all subjects, that voxel was included in the analysis. This measure was used because FA is more susceptible to errors arising from partial volumes [27], and this FA cut-off value allowed us to dissociate white matter structure from other tissue [28].

## 3. Results

As for psychological measures, the CAPS total score significantly recovered between post and followup (6.6 ± 11.2 to 1.6 ± 2.9, *P* < 0.05). Scores on STAI-state (44.1 ± 11.4 to 39.2 ± 10.4, n.s.), STAI-trait (42.7 ± 9.6 to 43.2 ± 11.2, n.s.), CES-D (12.1 ± 10.6 to 10.7 ± 9.3, n.s.), Rosenberg self-esteem scale (32.8 ± 8.2 to 32.8 ± 8.9, n.s.), and PTGI-J (33.8 ± 18.9 to 34.3 ± 19.3, n.s.) were not significantly changed from post to followup (Table 1).

**Figure 1:**
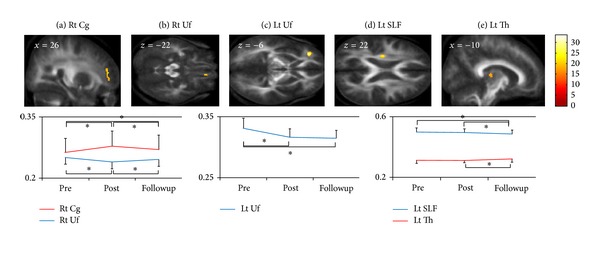
(a) FA in the right anterior Cg was significantly increased from pre to post (*P* < 0.05, paired *t*-test) and from pre to followup (*P* < 0.05, paired *t*-test), but it was significantly decreased from post to followup (*P* < 0.05, paired *t*-test). (b) FA in the right Uf was significantly decreased from pre to post (*P* < 0.05, paired *t*-test), but it was significantly increased from post to followup (*P* < 0.05, paired *t*-test). (c) FA in the left Uf was significantly decreased from pre to post (*P* < 0.05, paired *t*-test) and from pre to followup (*P* < 0.05, paired *t*-test). (d) FA in the left SLE was significantly decreased from pre to followup (*P* < 0.05, paired *t*-test) and from post to followup (*P* < 0.05, paired *t*-test). (e) FA in the left Th was significantly increased from post to followup (*P* < 0.05, paired *t*-test). These FA changes are illustrated by the plots at the bottom: vertical axes represent FA at peak voxels in each cluster, and horizontal axes indicate time periods. Error bars represent standard deviations. Colored bars represent *F* values. FA: fractional anisotropy; Rt: right; Lt: left; Cg: cingulum; Uf: uncinate fasciculus; SLE: superior longitudinal fasciculus; Th: thalamus.

**Figure 2:**
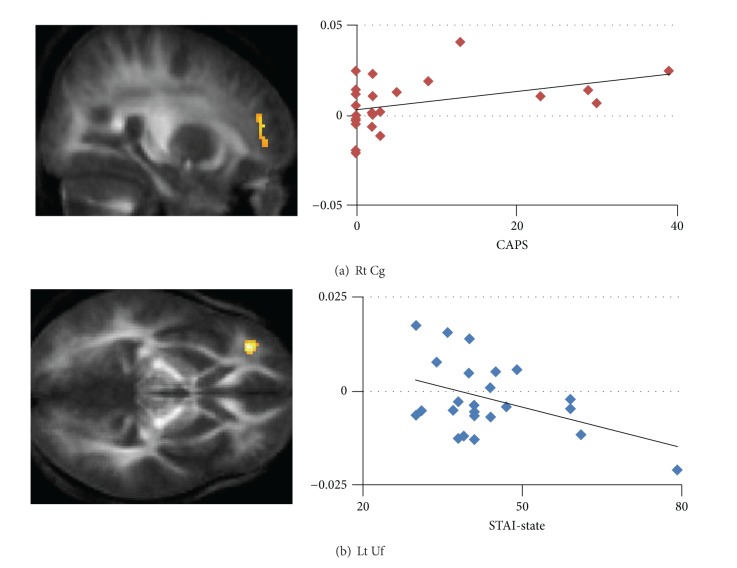
(a) CAPS scores were positively associated with FA changes from pre to followup in the right anterior Cg (Spearman's Rho = 0.414, *P* = 0.039), and (b) STAI-state scores were positively associated with FA changes from post to followup in the left Uf (*r* = −0.440, *P* = 0.028), as illustrated by the scatter plots on the right. Vertical axes represent FA changes at peak voxels in each cluster, and horizontal axes indicate (a) CAPS scores and (b) STAI-state scores. FA: fractional anisotropy; Rt: right; Lt: left; Cg: cingulum; Uf: uncinate fasciculus.

We found differential FAs to be a significant main effect of time period (pre/post/followup) in the right anterior Cg, bilateral Uf, left superior longitudinal fasciculus (SLF), and the thalamus (Table 2, Figure 1). *post hoc* correlation analyses revealed a significant positive correlation between the FA changes in the right anterior Cg from pre to followup and CAPS scores at post (Spearman's Rho = 0.414, *P* = 0.039, Figure 2(a)) and a significant negative correlation between the FA changes in the left Uf from post to followup and STAI-state scores at post (*r* = −0.440, *P* = 0.028, Figure 2(b)).

## 4. Discussion

To the best of our knowledge, this is the first longitudinal study to track microstructural changes in the brain at three time points: before, a short time after, and a long time after a disaster. We found differential FA at each time point in the right anterior Cg, bilateral Uf, left SLF, and left thalamus. According to the results of additional comparisons, we categorized the data according to the following three types of FA changes: normalization from initial FA changes in the right anterior Cg and right Uf (Figures 1(a) and 1(b)), sustained FA changes from the early phase in the left Uf (Figure 1(c)), and FA changes appearing during the late phase in the left SLF and thalamus (Figures 1(d) and 1(e)).

Increased or decreased WMI both a short and a long time after a disaster is likely to be due to synaptic enhancement and shrinkage, respectively. Biologically, synaptic enhancement or shrinkage has been observed in altered white matter following stress [3]. These changes are caused by hyper-secretion of glucocorticoids, a stress hormone [29]. The effects of stress hormones on the brain are observed as an inverse U shape, depending on dose and time [30]. Additionally, stress-induced structural and functional alterations have been shown to be reversible, at least in the prefrontal cortex [31, 32]. In the context of these considerations, we assumed that FA changes in the right anterior Cg were consistent with the aforementioned concept and that increased FA in the thalamus and decreased FA in the Uf and SLF reflected the rising and falling components, respectively, of the inverse U-shaped curve that characterizes such changes.

The results of correlation analyses and our previous findings [10] led us to speculate that, in some subjects, the WMI changes reflected normalization after initial changes. Such reversible WMI changes are congruent with the aforementioned biological conceptualizations [30–32]. As discussed below, we interpreted such WMI changes as not only signs of recovery from emotional distress shortly after a disaster but also as predictors of a better prognosis for subjects with more pronounced psychological responses to a stressful event, namely, following two cases.

First, the WMI changes in the left Uf identified in some subjects who reported distress indicated that recovery from emotional distress is possible following a stressful event. Our previous study demonstrated that the WMI was greater in the left Uf after the earthquake as compared with before the earthquake and was positively correlated with state anxiety levels, suggesting that the increased WMI in the left Uf was an acquired sign of emotional distress soon after a disaster [10]. In the present study, the WMI in the left Uf decreased from soon after (Post) to one year after (followup) the earthquake in subjects who had had higher state anxiety levels soon after the earthquake (Post). The Uf, which is also involved in emotional processing [33], is a principal white matter tract that connects the orbitofrontal cortex (OFC) and limbic regions, including the amygdala and the anterior temporal cortices [34, 35]. Neural responses in the OFC are preferentially enhanced, along with those in the amygdala, during extinction [36] and this relationship is crucial to the voluntary regulation of emotion [37]. Taking the functional roles of the Uf into account, the current results suggest that WMI in the Uf, which was elevated soon after the earthquake, reflecting the requirements of emotional regulation related to postearthquake stress, declined 1 year after the earthquake.

Next, the WMI changes in the right anterior Cg in some subjects who reported subclinical PTSD symptoms also suggested that a stressful event would strengthen structural connectivity, particularly in vulnerable subjects. The anterior Cg bundle is a part of the principal white matter tract in the Papez circuit, which includes the ACC and the amygdala [38]. Reduced WMI in the anterior Cg is frequently reported in patients with anxiety disorders such as PTSD [6–8, 39], social anxiety disorder (SAD) [40], and generalized anxiety disorder (GAD) [41] and in healthy subjects with high trait anxiety [42, 43]. It has been suggested that reduced WMI in the Cg represents dysfunctional emotion processing in such patients [6–8, 39–41]. Our previous study revealed that lower WMI in the right anterior Cg was a preexisting vulnerability factor for emotional distress soon after a disaster [10]. The current results showing the positive correlation between increased WMI in the right anterior Cg and CAPS scores demonstrated that those who had more PTSD symptoms soon after the earthquake displayed increased structural connectivity in the anterior Cg from before to 1 year after the earthquake. Furthermore, although depression and anxiety levels did not improve from 3-4 months after to 1 year after the earthquake, none of the subjects in this study developed clinical PTSD. Together, the findings suggest that dynamic WMI changes in the Cg predict a better prognosis, whereas persistently lower WMI represents cognitive dysfunction, implying the development of anxiety disorders (e.g., PTSD, SAD, and GAD).

White matter changes due to maturation and/or aging should be taken into account when interpreting the results, because this study did not include a control group, which is a limitation of this study. This is particularly problematic with respect to interpreting WMI changes, such as the increased WMI in the thalamus and the decreased WMI in the SLF, without evaluating their correlation with psychological measures. A recent study that investigated WMI changes due to maturation and/or aging revealed that peak FAs in the Cg, Uf, and SLF were observed in subjects older than the age range of our subjects (19 to 25 yr) [44]. Another study reported increased FA in thalamic radiations with age [45]. In contrast, another recent study investigating longitudinal FA changes at younger ages found that FA in the Uf decreased by almost half in subjects ranging in age from 19 to 25 [46]. Thus, decreased WMI in the SLF is unlikely to have occurred in our subjects, whereas it is difficult to reject the possibility that our finding of the increased WMI in the thalamus is a result of maturation. Nevertheless, the interpretation of WMI changes, such as the increased WMI in the Cg and the decreased WMI in the Uf, and their correlation with psychological measures are less problematic. We believe that the current study provides sufficient evidence of the short- and long-term effects on the brain microstructure despite the absence of a control group.

## 5. Conclusions

The present followup DTI study showed the long lasting effects of stressful events on brain microstructure. Our findings suggest that microstructures within the brain change due to stress and recovery. We assumed that brain microstructural changes due to stressful life events were not static but dynamic through life. Recently, the alteration of functional and structural connectivity, including regions adjacent to the Cg and the Uf, was reported in subjects soon after a disaster [47, 48]. Therefore, further longitudinal investigations using multimodal approaches are necessary to examine whether the stress-induced alterations in brain structure are reversible.

## Figures and Tables

**Table 1:** Psychological measures.

	Post	Followup	*P* value
CAPS (total)	6.6 ± 9.6	1.6 ± 2.9	0.04
CES-D score	12.1 ± 10.6	10.7 ± 9.3	n.s.
STAI scores			
State	44.1 ± 11.8	39.2 ± 10.4	n.s.
Trait	42.7 ± 9.6	43.2 ± 11.2	n.s.
Self-esteem	32.8 ± 8.2	32.8 ± 8.9	n.s.
PTGI-J (total)	33.8 ± 18.9	34.3 ± 19.3	n.s.

Values are shown as mean ± standard deviation.

CAPS: clinician-administered PTSD scale, CES-D: center for epidemiologic studies depression scale, STAI: state-trait anxiety inventory, and PTGI-J: Japanese version of the posttraumatic growth inventory.

**Table 2:** MNI coordinates, voxel sizes, *F* values, and *P* values for results of the SPM analyses.

Brain region	MNI coordinates			*k* (voxels)	*F* values	*P* values (FWE)
	*x*	*y*	*z*			
Rt anterior Cg	26	52	14	55	21.68	0.002
Rt Uf	8	46	−22	10	19.38	0.007
Lt Uf	−32	44	−6	72	33.49	0.000
Lt SLF	−28	−18	22	63	24.21	0.001
Lt thalamus	−10	−22	10	23	17.96	0.017

MNI: montreal neurological institute, Rt: right, Lt: left, Cg: cingulum, Uf: uncinate fasciculus, and SLF: superior longitudinal fissure.
